# Effect of Variations in Aggregate Ratios on the Fresh, Hardened, and Durability Properties of Self-Compacting Concrete

**DOI:** 10.3390/ma17225639

**Published:** 2024-11-18

**Authors:** Yahya Kaya, Hatice Elif Beytekin, Ali Mardani

**Affiliations:** 1 Department of Civil Engineering, Bursa Uludag University, Bursa 16059, Turkey; 512126007@ogr.uludag.edu.tr; 2 Department of Architecture, Bursa Uludag University, Bursa 16059, Turkey; 511712002@ogr.uludag.edu.tr

**Keywords:** self-compacting concrete, coarse aggregate ratio, fresh state properties, compressive strength, modulus of elasticity, durability

## Abstract

Self-compacting concrete (SCC) is a type of concrete that can be poured into complex geometries and dense reinforcement areas without the need for mechanical vibration, exhibiting excellent segregation resistance and flowability. Its adoption in the construction industry has surged in recent years due to its environmental, technical, and economic advantages, including reduced construction time and minimized occupational hazards. The performance of SCC is significantly influenced by the properties of the aggregates used. This study investigates the effects of variations in the coarse-to-fine aggregate ratio and water/binder (w/b) ratio on the fresh, hardened, and durability properties of SCC. A total of eight different SCC mixtures were prepared, utilizing two distinct s/b ratios and four varying fine-to-coarse aggregate ratios. The results indicated that increasing the s/b ratio enhanced fresh state performance but adversely affected mechanical strength and shrinkage behavior. Furthermore, the need for admixture and flow times improved with increasing coarse aggregate content, attributed to the reduction in cohesiveness and viscosity. However, this change did not significantly impact mechanical properties, while high-temperature resistance and shrinkage exhibited an upward trend.

## 1. Introduction

Greenhouse gas emissions, air pollution, and climate change are among the main factors leading to global issues. In the process of addressing these problems, topics like developing alternatives to products that emit CO_2_ during production, renewable energy sources, and energy efficiency have gained importance [1]. Globally, the primary source of greenhouse gases is the use of fossil fuels, with cement production holding the largest share when categorized by purpose.

For these reasons, the sustainable development of concrete has attracted widespread interest in the advancement of concrete technology. In this context, self-compacting concrete (SCC), one of the greatest innovations, is known to have clear advantages in terms of reducing construction costs and improving the construction environment compared to conventional vibrated concrete. This makes SCC an important step toward the development of sustainable concrete [1,2,3].

SCC is defined as concrete that can spread into a mold, fills limited areas in complex shapes, includes dense reinforcements, compresses only under its weight during the casting process without the need for mechanical vibration, and does not show segregation or bleeding [1,2]. In addition, it is known that self-compacting concrete has significant environmental, technical, and economic advantages such as reducing cost, occupational accidents, and construction time [3,4]. The use of SCC in the construction industry has increased significantly in recent years [5].

It was emphasized that the strength, shape, size, surface texture, and usage rate of the aggregates used in self-compacting concrete mixtures significantly affect concrete’s fresh and hardened state properties [6,7]. It was reported that void structures can form in self-compacting concrete that does not have the correct usage rate [8]. Unlike traditional concrete, the use of powder (filler) material was suggested by many researchers [9] to increase the amount of fine material in self-compacting concrete. In addition, it was emphasized that the rheological properties, fresh state fluidity, and weathering resistance of the SCC matrix are greatly affected by the ratio of fine to coarse aggregate and the powder material [10,11]. In addition, it was understood that the dough volume is increased by reducing the aggregate volume and adding different powder substances to ensure high flow ability [12]. However, it is known that as the fine material content increases, the sensitivity to shrinkage cracks increases.

The results of previous studies have shown that the compressive strength increase rate of SCC at early ages is higher than that of conventional concrete. It was emphasized that this is due to the presence of powder material and the retention ability of water in the SCC [13]. On the other hand, due to the presence of powder material and the absence of external and internal vibration, the ITZ in SCC is stronger than conventional concrete, and hence the compressive strength of SCC for a given water/cement ratio is higher than that of conventional concrete [14]. Different researchers have stated that the compressive strength of concrete depends on the cement/aggregate ratio and the aggregate size as well as the w/c ratio [15,16].

In a study conducted by Parra et al. [13], the mechanical properties of SCC in the medium and low strength range were investigated. For this purpose, by examining SCC mixtures with a water/cement ratio of 0.45–0.65, it was reported that the tensile strength was 15% lower than conventional concrete. Su et al. [17] showed that decreasing the coarse aggregate ratio compared to the total aggregate ratio did not change the modulus of elasticity of SCC [18]. Analyzing results reported by other researchers, Domone [18] showed that at low strength levels, the modulus of elasticity of SCC was 40% lower than conventional concrete, while at higher strength levels, this value was limited to 5%. This behavior was attributed to the lower coarse aggregate content in SCC compared to conventional concrete.

The usage rates of SCC mixtures were investigated by various authors. Other studies on the subject are summarized in Table 1.

According to the general conclusions that can be drawn from the studies summarized in Table 1, it was understood that the maximum grain size of coarse aggregate (dmax) and the fine/coarse aggregate ratio in self-compacting concrete mixtures significantly affect the mechanical properties and workability of concrete. It was reported that, by increasing the size of coarse aggregate, the fracture energy and the modulus of elasticity of concrete generally increase, and better strength is achieved with lower water/cement ratios. However, it was emphasized that an excessive increase in the coarse aggregate ratio may have a negative effect on mechanical performance [11]. In addition, increasing the fine/coarse aggregate ratio increases the fluidity and compactness of fresh concrete, while also positively affecting surface roughness and bond strength. However, this may negatively affect the durability of concrete due to the dominant role of coarse aggregate in mechanical properties. For these reasons, for optimum performance, it is recommended to use coarse and fine aggregates in balanced proportions and select appropriate water/cement ratios.

Although there are many standards in effect, it was determined that there is no definitive procedure for determining the optimum mixing ratio for SCC. It is understood from the table that limited and contradictory results were obtained regarding the effect of the aggregate size distribution used on the fresh and hardened state properties of SCC. In addition, it was observed that the studies are generally limited to the investigation of the workability and mechanical properties of self-compacting concrete mixtures.

For this reason, it was understood that new research is necessary regarding the effect of the aggregate type, size, and usage rate on the properties of mixtures. In this study, the effect of the coarse/fine aggregate ratio in SCC mixtures on fresh state properties and compressive strength performance, the modulus of elasticity, high-temperature resistance, and drying shrinkage behavior was investigated. Various macroscopic properties of concrete mixtures were examined by combining microscopic SEM analyses.

## 2. Materials and Methods

### 2.1. Materials

In this study, CEM I 42.5 R-type cement and F class fly ash were used as binders. The chemical composition and some physical and mechanical properties of the cement and fly ash obtained from the manufacturer are shown in Table 2.

To provide the desired dispersion values in concrete mixtures, a single polycarboxylate-ether-based high water-reducing admixture was used at different usage rates. Some properties of the water-reducing admixture used, given by the manufacturer, are shown in Table 3.

**Figure 1:**
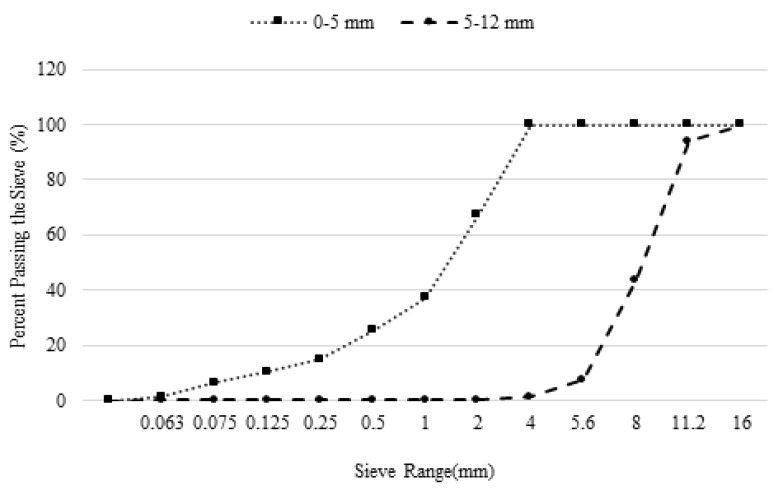
Gradation curves of aggregates.

Crushed limestone aggregate was used as coarse and fine aggregate in the mixtures. The specific gravity and water absorption capacities of the aggregates used, determined according to EN 1097-6 standard [23], were determined as 2.63–2.73 and 3.7–4, respectively. Sieve analyses of the aggregates used in the mixtures were performed according to the EN 206 standard [24] and are shown in Figure 1. As stated in the standard, materials passing through a sieve with a mesh size of 0.063 mm are considered powder. The powder material used within the scope of the study was obtained by sieving the limestone that was used as fine aggregate. The amount of powder in the mixtures was kept constant.

### 2.2. Preparation of Mixtures

Within the scope of the study, two series of concrete mixtures with different water/binder ratios were prepared. Coarse aggregate (5–12 mm) was used at rates of 15%, 30%, 45%, and 60% of the aggregate amount for each series of concrete mixture. In all concrete mixtures, in the first stage, the cement dosage, fly ash dosage, dust amount, and spreading values were kept constant at 400 kg/m^3^, 100 kg/m^3^, 120 kg/m^3^, and 65 ± 2 cm, respectively. Material quantities for a 1 m^3^ concrete mixture are given in Table 4. The naming of the mixtures was made according to the w/c ratio and coarse aggregate content. For example, the w/c ratio was 0.4, and the mixture containing 15% coarse aggregate was named 0.40–15.

### 2.3. Method

The pan type of concrete mixer was used in the production of mixtures. The preparation of concrete mixtures in the mixer was carried out following the EN 14845-1 stanard [25]. According to the standard in question, aggregate materials were added to the mixer and mixed. Then, binding materials were added, and dry mixing was completed for 1 min. Water was added to the dry mixture and mixed for another minute, and a high-level water-reducing additive was added to the remaining mixture and mixed for another minute. The mixing time for all mixtures was kept constant at 3 min in total.

The aim of the spreading test was to observe the flow ability of fresh SCC and to measure the diameter that the sample would create by spreading with its own weight. The spreading test was carried out with a traditional Abram’s cone, and the horizontal spreading distance was measured. The test was carried out in accordance with the EN 12350-8 standard [26]. After the surface of the spreading table placed on a completely flat surface was moistened, Abram’s cone was filled with concrete to its center. Then, the cone was pulled up perpendicularly to the table, and the time for the concrete to reach the 50 cm in diameter, previously marked on the table (T50), and the final spreading diameter was measured in two perpendicular directions, and the test was completed. The V-funnel test of the mixtures was carried out using a 5 × 5 cm aperture with a 10 lt capacity V-shaped funnel. The V-funnel time was determined by measuring the emptying time of the funnel. The test was carried out by the EN 12350-9 standard [27] in order to examine the ability of KYB to pass through a narrow section under its own weight. The unit weight of fresh concrete is determined according to EN 12350-6 [28].

**Figure 2:**
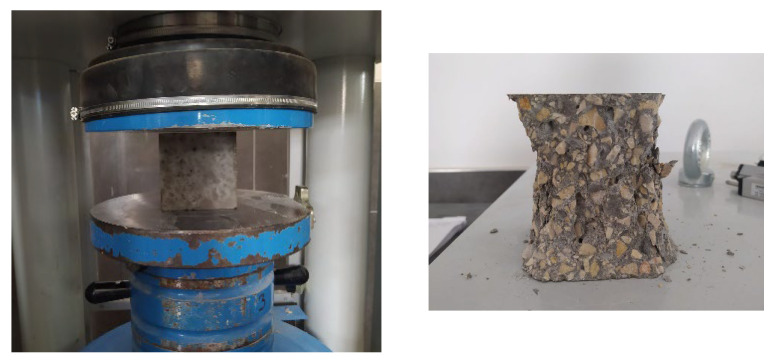
The application of the concrete compressive strength test.

The compressive strength test of the samples was carried out on 100 mm cube samples by EN 12390–3 standard [29]. While preparing the samples, the inner surface of the molds was lubricated before the fresh concrete was placed in the molds. The samples were placed in the lubricated molds. The samples were placed in the middle of the appropriate press machine and tested with a load of 13.5 KN per second. The determination of the compressive strength of the samples and the image of the broken sample are shown in Figure 2.

The modulus of elasticity test was tested on cylindrical samples with a diameter of 10 cm and a height of 20 cm. Firstly, to smooth the surfaces of the cylindrical samples, the sample surface was shaved on a smoothing grinding machine. Then, a control sample was selected and subjected to a pressure test, and its strength was determined. Then, 40% of the obtained force was applied to the other two samples in a load–unload manner, and axial stress–strain values corresponding to the calculated loads were obtained with the help of the compactor on the frame placed around the sample.

The length change in the concrete mixtures due to drying shrinkage was determined on 75 × 75 × 285 mm prismatic samples in accordance with the ASTM C157/C157M − 17 standard [30]. For this purpose, the samples were removed from the mold 24 h after casting and cured in water with 95 ± 5% relative humidity and 23 ± 2 °C temperature for 48 h. Then, they were removed from the curing pool and kept in a curing cabin with a temperature of 20 °C and a relative humidity of 55% for 28 days, and length change measurements were taken on certain days. The length change in the samples for 28 days was calculated as shown in Equation (1).

S = (L_1_ − L)/L_0_ × 100 (1)

Here, S represents the length change percentage of the sample, L_1_ represents the initial length measurement value of the sample removed from the curing pool, L represents the length measurement value of the sample on the following days, and L_0_ represents the effective measurement length of the sample.

The water absorption capacity of concrete mixtures was calculated according to the ASTM C642-97 standard [31] on 100 mm cube samples.

**Figure 3:**
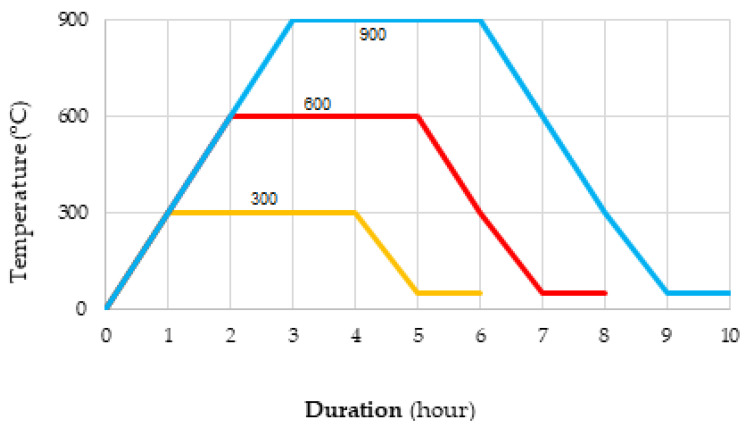
High-temperature resistance test process.

In this study, the resistance of SCC samples to high temperatures was investigated. After curing in water for 28 days, the samples were kept in a rotary air oven at 105°C for 24 h. Then, they were exposed to temperatures of 300, 600, and 900 °C. While determining the temperatures, the previous studies of the authors were taken into account. In all mixtures, the time to reach these temperatures was kept constant at 5 °C/minute and the waiting time at 180 min (Figure 3). The weight change, surface damage, strength, and permeability properties of the samples exposed to the mentioned temperature were determined. The obtained results were compared with the results of the samples not exposed to high temperatures.

The images of the samples cured for 28 days were taken with the help of a scanning electron microscope (SEM) to examine their morphology. To this end, small samples were taken from the samples (approximately 1 cm^2^) and kept in the oven at 105 degrees for one day. Then, the surface of the dry samples was coated, and SEM images were taken.

## 3. Results

### 3.1. Fresh and Hardened State Unit Weight

**Figure 4:**
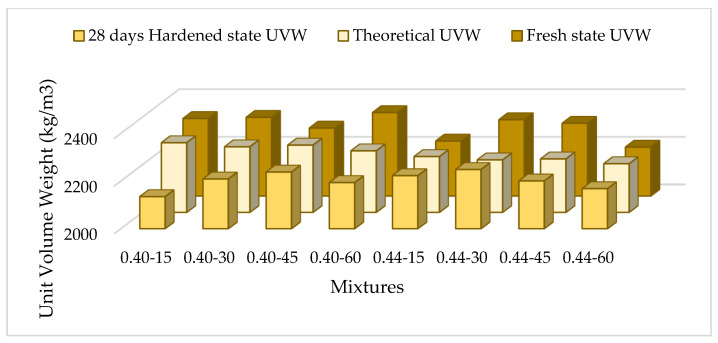
Fresh and hardened state unit weights of the mixtures.

Fresh and hardened state unit weights of the mixtures are shown in Figure 4.

As shown in the figure, the hardened unit weight values decreased by 2% to 8% compared to the fresh BHA values, indicating minimal variation. In mixes with a 0.40 s/b ratio, no notable change was observed between the fresh and hardened unit weights as the coarse aggregate content increased. Similarly, for mixtures with a 0.44 s/b ratio, the fresh and hardened unit weights remained relatively constant despite the rise in the coarse aggregate proportion.

### 3.2. Fresh State Result

Fresh state tests were conducted to assess the impact of the coarse-to-fine aggregate ratio on the filling ability, flowability, and viscosity of self-compacting concrete mixtures. Table 5 presents the water-reducing admixture requirements, spread values, T50, and V-funnel flow times for achieving the target spread value of 65 ± 2 cm in the mixtures.

Table 5 indicates that the T50 results, representing the time for mixtures to reach a 500 mm diameter in the slump flow test, ranged from 3 to 7 s, meeting the SCC requirements as per EN 206-12 standards [32,33]. The influence of coarse aggregate content on concrete flowability was observed. For mixtures with a consistent coarse/fine aggregate ratio, an increase in the water/binder ratio led to enhanced fluidity, reduced viscosity, and shorter T50 times, as expected. Additionally, T50 times decreased as the coarse aggregate content increased. Previous studies in the literature have highlighted that increasing the fine aggregate content raises the total surface area, reducing the free water and negatively affecting workability performance [34,35], consistent with findings from Faraj et al. [35] and EFNARC [32].

In a study by Khaleel et al. [21], SCC mixtures included crushed gravel, unbroken gravel, and crushed limestone aggregates with maximum sizes of 10 mm and 20 mm. It was observed that increasing the maximum aggregate size directly reduces the fluidity and passing ability of SCC. When unbroken gravel, which has a smoother surface structure, was used, fluidity, passing ability, and resistance to weathering increased. The study also noted that water adsorption by fine aggregate particles and increased friction between aggregate particles raised the viscosity of the mixture.

It Is well-established that V-funnel flow time correlates directly with the viscosity and cohesiveness of mixtures. Regardless of the water/binder ratio, V-funnel times, which indicate segregation resistance and concrete’s ability to flow out of the funnel, followed a similar trend as coarse/fine aggregate ratios increased. It was found that increasing the coarse/fine aggregate ratio up to 45/55 enhanced flowability and decreased V-funnel times. However, mixtures with ratios exceeding 45/55 exhibited reduced spread performance and longer V-funnel durations. Similar observations were reported by Tawfik et al. [36]. Khaleel et al. [21] also noted that, in mixtures with higher fine aggregate content, mixed water tends to adhere to fine particles, increasing friction between aggregates, thereby raising viscosity. Lin et al. [11] pointed out that low viscosity prevents coarse aggregate coating, causing it to settle and hindering flow. This mechanism likely explains the spread results and V-funnel values in mixtures with coarse/fine ratios above 45/55. The optimal coarse/fine aggregate ratio for fresh state properties was identified as 45/55.

### 3.3. Compressive Strength, Modulus of Elasticity, and Poisson’s Ratio

#### 3.3.1. Compressive Strength

Table 6 presents the 28-day compressive strength, modulus of elasticity, and Poisson’s ratio values of the mixtures.

In all SCC mixtures, a significant decrease in compressive strength was observed as the water/binder ratio increased. However, the highest compressive strength was obtained in mixtures containing 30% coarse aggregate. In mixtures where the volume of coarse aggregate increased from 15% to 60% of the total aggregate content, there was an initial slight decrease in compressive strength, followed by an improvement. This observation can be attributed to the properties of concrete as a composite material; since aggregates are generally stronger than cement paste, an increase in the aggregate content enhances the overall strength of concrete. However, the increase in aggregate volume also leads to the expansion of the cement–aggregate ITZ, which is weaker due to its higher porosity and lower strength, potentially resulting in a loss of strength. The thickness of the ITZ, typically between 30 and 50 μm, increases as the volume of coarse aggregate rises, shortening the distance between aggregates and negatively affecting the compressive strength. According to composite theory, while the increase in aggregate content improves strength, the expanding ITZ volume counterbalances this improvement, leading to an irregular change in the compressive strength.

Studies in the literature have shown that the properties of the ITZ are largely dependent on factors such as aggregate size, volume, shape, and surface texture [37]. Şahin et al. [38] indicated that the size, shape, and surface texture of aggregates have significant effects on the ITZ and influence the cracking behavior of concrete. Torrijos et al. [39] noted that larger aggregates could inhibit crack growth and cause cracks to deflect or branch. It was reported that decreases in the ITZ strength significantly increase the fracture energy (GF), thus increasing the resistance of concrete to crack propagation [40,41,42].

Studies examining the effect of the maximum aggregate size (dmax) on the mechanical properties of concrete have generally shown that GF increases with dmax. Researchers such as Zhao et al. [41] have reported a strong relationship between GF and dmax, indicating that larger aggregate sizes increase GF and enhance the fracture resistance of concrete. Issa et al. [43] observed that GF increases monotonically with the increase in dmax, linking this to the strengthening of the ITZ by larger aggregates, which increases energy absorption capacity during crack propagation.

In numerous studies, the effects of coarse aggregates on the mechanical behavior of concrete were examined, and it was reported that optimal coarse aggregate proportions enable the achievement of high compressive strength [44,45,46,47]. Concrete strength is influenced by various factors, such as the s/b ratio, paste–aggregate bonding, the cement-to-aggregate ratio, and the aggregate size [48,49]. Considering the interdependence of these variables, the specific effect of aggregate size on the mechanical properties of concrete has not been definitively determined [7,50]. Moreover, it was reported that the distribution of the aggregate size can have varying effects depending on the strength class of the concrete [47]. Improvements in strength performance are generally attributed to advancements in the paste phase [34], while strength loss is linked to a weaker ITZ [7].

Studies by Vu et al. [48], Akçaoğlu et al. [51], and Rao and Prasad [42] observed a slight increase in compressive strength with larger aggregate sizes. Akçaoğlu [51] noted that, in high-strength concrete, an increase in the volume of coarse aggregates reduces the ITZ ratio due to the smaller surface area compared to finer aggregates. As a result, the increase in compressive strength was less pronounced compared to mixtures with finer aggregates. Therefore, under constant cement content and workability conditions, the effect of the aggregate size on the compressive strength may vary [52,53].

#### 3.3.2. Modulus of Elasticity and Poisson’s Ratio

**Figure 5:**
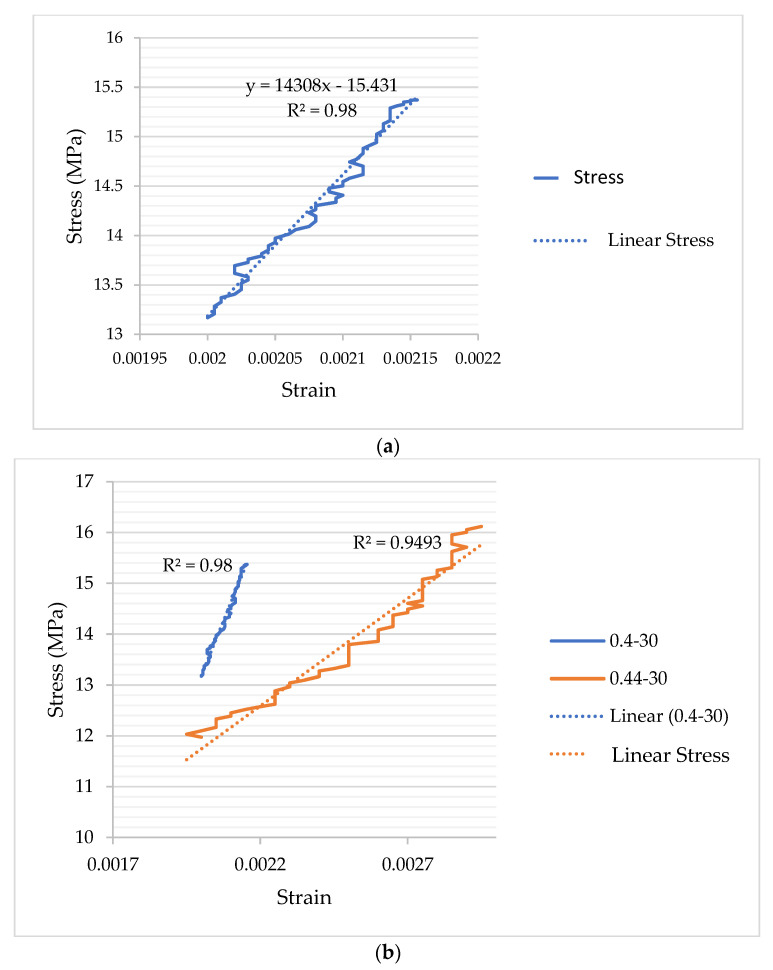
The load–deformation graphs. (**a**) The behavior of the 0.40–30 mixture. (**b**) Comparisons of mixtures containing 30% coarse aggregate at 0.44 and 0.40 s/b ratios.

Table 6 displays the load–deformation plots for mixtures with a 0.40 and 0.44% s/b ratio and 30% coarse aggregate content. The modulus of elasticity is a key parameter influencing various concrete properties. Aggregate size distribution plays a significant role in determining the modulus of elasticity. A well-balanced size distribution contributes to a more homogeneous concrete structure, enhanced load distribution, and, consequently, a higher modulus of elasticity. Numerous factors impact the modulus of elasticity in concrete, with the ITZ being one of the most critical. Load-deformation graphs of some selected mixtures are given in Figure 5.

The aggregate–paste interface represents the contact zone between the aggregate and cement paste, the two core components of concrete. The quality of this interface plays a crucial role in determining the overall behavior of concrete, including its modulus of elasticity. A robust aggregate–paste interface facilitates effective load transfer, minimizes the risk of cracking, and enhances strength. Conversely, a weak interface can lead to premature concrete deterioration, reduced strength, and a shorter service life.

The relationship between aggregate and the modulus of elasticity can be summarized as follows: (i) As the coarse aggregate volume increases, the void ratio in the concrete mix also rises, leading to a less dense structure and, consequently, a lower modulus of elasticity. (ii) Coarse aggregate particles help transfer applied loads through the cement paste; however, oversized or irregularly shaped aggregates can hinder this transfer, reducing the modulus of elasticity. (iii) The size and distribution of coarse aggregates impact concrete’s cracking behavior. Larger or irregularly shaped aggregates may induce stress concentrations, increasing the risk of cracking and negatively affecting the modulus of elasticity. (iv) The interaction between coarse aggregates and cement paste influences the concrete’s microstructure, where a weak aggregate–paste interface can diminish the modulus of elasticity.

Rao and Prasad [46] noted that in SCC, the modulus of elasticity increased slightly with larger coarse aggregates due to their higher stiffness. In contrast, Tasdemir et al. [45] reported a minor decrease in the modulus with increasing aggregate size.

As observed, increasing the coarse aggregate volume from 15% to 60% initially led to an increase and then a decrease in the modulus of elasticity, reflecting a pattern similar to changes in compressive strength. Meddah et al. [47] mentioned that, because the total aggregate volume remained constant, variations in the coarse-to-fine aggregate ratio did not significantly impact the modulus of elasticity.

Furthermore, the high strength of SCC generally results in ITZ stiffness approaching that of the matrix, which reduces the impact of the ITZ volume on the modulus of elasticity [54]. Studies by Wu et al. [55] and Meddah et al. [47] indicated that, when the paste volume is kept constant, the coarse-to-fine aggregate ratio has a minimal effect on the modulus of elasticity.

**Figure 6:**
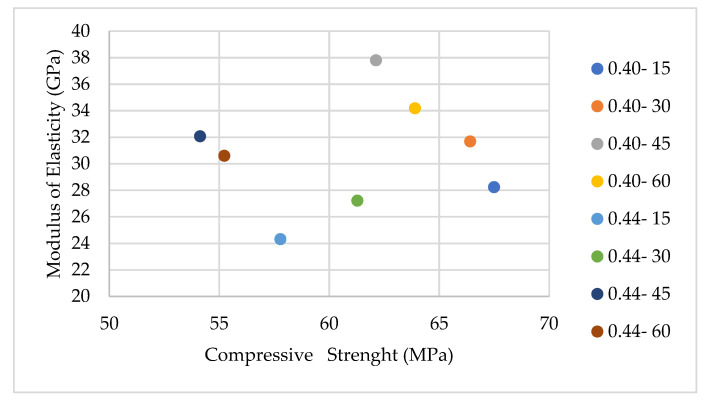
The relationship between the compressive strength and the modulus of elasticity of the mixtures.

The relationship between the compressive strength and the modulus of elasticity of the mixtures is shown in Figure 6.

The trends observed in the compressive strength and modulus of elasticity indicate that these properties are more pronounced at higher s/b ratios. In higher-strength concrete, increasing the aggregate size leads to a decrease in surface area, thereby narrowing the transition zone. However, the quality of the transition zone surrounding coarse aggregates is inferior compared to that around finer aggregates. Consequently, the increase in the compressive strength and the modulus of elasticity becomes negligible when finer aggregates are present.

Additionally, in SCC with lower s/b ratios, changes in tensile strength occur due to the differing ITZ structure and matrix properties compared to those with higher s/b ratios.

The trend in the mechanical properties does not show significant dependence on the coarse aggregate-to-total aggregate ratio. This is attributed to the two opposing effects of increasing aggregate volume: while it enhances strength and stiffness, it also increases the ITZ volume. The modulus of elasticity, in particular, exhibits larger variations (approximately 17%) because it is less influenced by the ITZ, resulting in a diminished stabilizing effect compared to other mechanical properties.

Given the higher paste volume in SCC relative to conventional concrete, it can be concluded that the properties of the paste phase exert a greater influence on the mechanical properties of PBM. Furthermore, for a constant paste volume, variations in the coarse-to-fine aggregate ratio have a minimal effect on the mechanical properties of SCC. This behavior aligns with findings from Parra et al. [13], while Malazdrewicz et al. [56] noted that SCC achieves approximately 90% of its 28-day hardness within the first 3 days due to its highly compacted matrix.

### 3.4. SEM Analysis Results

**Figure 7:**
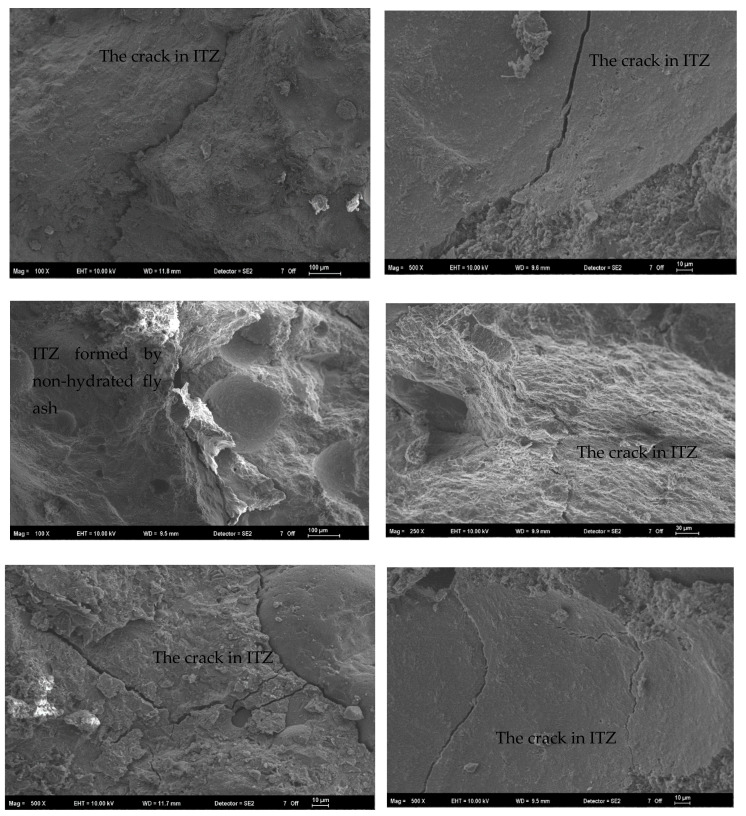
Images of cracks formed especially in the ITZ region of the samples.

Figure 7 displays the SEM images obtained from the mixtures.

As seen from SEM images, a weak zone is formed in the transition zone of the aggregate paste. It is known that these cracks are affected by parameters such as material incompatibility, curing conditions, and the w/c ratio. It is known that the ITZ is formed more especially in the use of coarse aggregate. The presence of the ITZ negatively affects the strength and durability performance.

### 3.5. High-Temperature Resistance

The strength and weight losses of the mixture after exposure to temperatures of 300 °C, 600 °C, and 900 °C are shown in Table 7.

At 300 °C, the compressive strength of SCC mixtures ranges from 94% to 105% relative to that at 25 °C (Table). The strength performance improves with the proportion of coarse aggregate, irrespective of the water/binder ratio, becoming more pronounced with increased coarse aggregate utilization and s/b ratio. When temperatures reach 250 °C, the water adsorbed in the C-S-H layers and capillary voids evaporates, causing volume shrinkage and layer adhesion [57]. Non-hydrated cement grains may hydrate at these temperatures, potentially increasing compressive strength [36]. However, if volume reduction surpasses the tensile strength of the cement paste, it can lead to cracking and strength loss [57].

Some studies have noted an improvement in the residual strength of SCC mixtures with increased porosity [58]. Biricik et al. [57] attributed strength increases between 150 and 300 °C to water migration in pores. Conversely, Pulkit and Adhikary [59] linked increased strength to the loss of bonds in silanol groups, resulting in shorter, stronger siloxane elements (Si-O-Si) with higher surface energies. This increase in strength is also associated with enhanced bonding properties of hydrates, where greater porosity leads to higher compressive strength [60].

Farez et al. [61] reported that the strength increase in SCC mixtures from 150 to 300 °C results from new hydration products’ filling porosity. However, these products do not sufficiently offset the porosity increase due to the cracking and dehydration of C-S-H. Thus, the strength improvement is mainly due to the hydration of anhydrous phases forming hydrates with superior bonding properties.

At 600 °C, the compressive strength performance of SCC mixtures decreases with higher coarse aggregate proportions, regardless of the water/cement ratio, and this effect is more evident at elevated s/b ratios. Ca(OH)₂, formed in the ITZ, is thought to accumulate more with increasing coarse aggregate. At 500 °C, Ca(OH)₂ loses chemically bound water, converting to CaO [54,57]. Alarcon-Ruiz et al. [62] suggested that, while the transformation of calcium hydroxide to lime and water vapor does not critically impact strength, internal stresses from vapor can damage the microstructure during cooling, with increased coarse aggregate potentially exacerbating these effects [59,63,64].

A strength loss of 80–85% is observed in all mixtures below 900 °C. At this temperature, the chemical water in CSH evaporates, impairing its binding properties [57]. Additionally, during the cooling phase after high-temperature exposure, CaO in cementitious systems reacts with moisture and CO₂, forming Ca(OH)₂ and CaCO₃, resulting in approximately a 40% volume expansion and subsequent disintegration.

### 3.6. Drying Shrinkage

**Figure 8:**
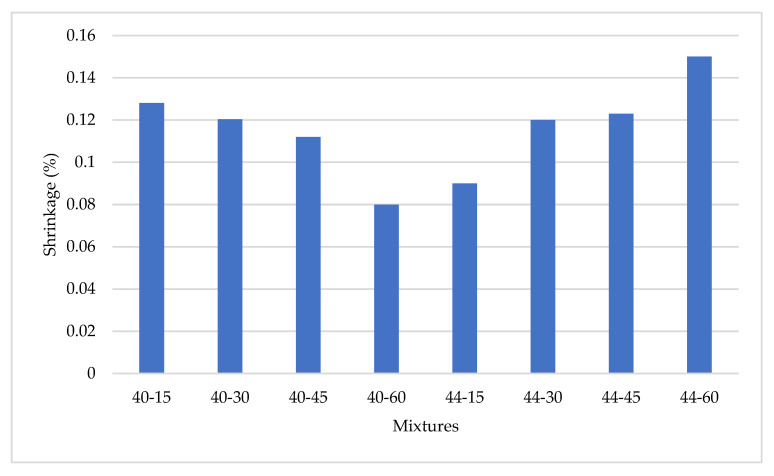
The 28-day shrinkage amounts of the mixtures.

The 28-day shrinkage amounts of the mixtures are shown in Figure 8.

It was observed that shrinkage increases from 0.09 to 0.15 with higher coarse aggregate content in mixes with a 0.44% water/binder ratio. Conversely, in mixes with a 0.40% water/binder ratio, shrinkage decreases from 0.128 to 0.08 as the coarse aggregate content rises. In SCC mixtures, a high proportion of coarse aggregate coupled with low fine aggregate content slows the rate at which water reaches the concrete surface. This phenomenon reduces evaporation and, consequently, shrinkage [65]. Additionally, it was noted that the amount of coarse aggregate is more influential than fine aggregate in enhancing internal curing and limiting dimensional changes due to the interfacial transition zone (ITZ).

However, the behavior varies with the s/b ratio. With a high s/b ratio, an increase in coarse aggregate content correlates with greater shrinkage, whereas at low s/b ratios, it tends to reduce shrinkage behavior.

In a study conducted by Zhu et al. [66], the effects of coarse aggregate volume and gradation on the drying shrinkage of SCC were examined. The study reported that the workability and drying shrinkage of SCC decrease with increasing coarse aggregate volume. The optimum coarse aggregate volume was determined to be 33%. The shrinkage was found to decrease as the volume ratio of 20 mm aggregate increased in mixtures with optimum coarse aggregate gradation (5–10 mm: 10–16 mm: 16–20 mm = 30%: 30%: 40%). This reduction in shrinkage was attributed to the better limiting effect of the coarse aggregate.

Bian et al. [67] reported that the superior water storage capacity of coarse aggregates compared to fine aggregates results in slower moisture diffusion into the cement paste and drying environment. Consequently, the excessive removal of internal moisture derived from aggregates in mixes with low s/b ratios leads to increased shrinkage.

During the water dispersion process, moisture in the SCC gradually decreases, creating a moisture gradient between the cement paste and coarse aggregates [68]. This gradient encourages the movement of water from the aggregates to the cement paste and ultimately to the environment. Due to the high water content retained in coarse aggregates, this diffusion process persists longer, intensifying shrinkage and prolonging its occurrence. Thus, water retention by coarse aggregates and high internal water content emerge as critical factors contributing to drying shrinkage in SCC. The higher water absorption rate of coarse aggregates, along with the substantial amount of moisture they retain, plays a significant role in the rapid development of drying shrinkage in SCC [58]. Additionally, intrinsic defects in coarse aggregates may reduce their ability to limit shrinkage in concrete, leading to more pronounced shrinkage [67].

**Figure 9:**
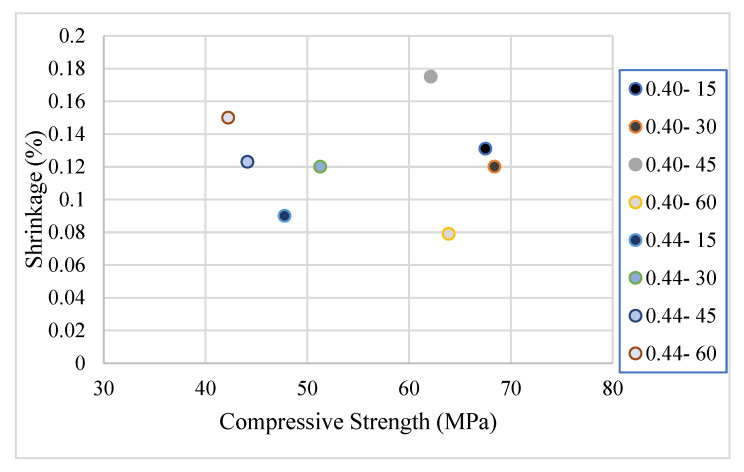
Relationship between compressive strength and shrinkage.

The relationship between compressive strength and shrinkage is given in Figure 9.

As can be seen from the figure, the effect of the aggregate content on shrinkage behavior changes according to the strength class. In mixtures with a high strength class, the amount of shrinkage decreases with the increase in the coarse aggregate content. On the other hand, in mixtures with lower strength, the amount of shrinkage increases with the coarse aggregate content. It is thought that this situation is due to the synergistic relationship between the performance of the dough phase and the strength performance of the aggregate phase.

## 4. Conclusions

In this study, the influence of the coarse-to-fine aggregate ratio and the influence of the variation in the s/b ratio on the fresh state, mechanical properties, and certain durability characteristics of SCC were thoroughly investigated. The key findings from the experiments and analyses are summarized below:

- Fresh state properties, including the need for the admixture to achieve target spreading, t50 time, spreading, and V-funnel transition time, improved with increasing s/b ratios, whereas the compressive strength, modulus of elasticity, high-temperature resistance, and shrinkage performance decreased.
- An increase in the proportion of coarse aggregates led to improvements in the need for additives for target spreading, t50, and V-funnel flow times, attributed to reduced viscosity resulting from decreased cohesiveness in the presence of coarse aggregates.
- No significant changes were observed in the 28-day compressive strength, modulus of elasticity, and Poisson’s ratio values for mixtures with increased coarse aggregate content.
- Increased high-temperature resistance and shrinkage were noted in mixtures with a higher proportion of coarse aggregates.

It is recommended that future studies explore the effects of changes in aggregate ratios on impact and abrasion resistance.

It is recommended that future studies investigate the effects of changes in aggregate ratios on impact and abrasion resistance.

In addition, ITZ can be further strengthened in the future by using different pozzolans and fibers.

## Figures and Tables

**Table 1:** Some studies on SCC.

Ref	Coarse Aggregate	Dmax	Fine Aggregate	Fineness Module	W/C	Highlights
[5]	limestone	9.5 12.5 19	limestone powder	2.85	0.38 0.53	It was emphasized that the fracture energy increased significantly with the increase in coarse aggregate dmax. It was reported that concrete mixtures with lower water/cement ratios and coarser aggregates have better strength.
[7]	limestone	9.5 12.7 19	limestone powder	2.85	0.38 0.53	It was reported that with the increase in coarse aggregate dmax from 9.5 mm to 19 mm, the compressive strength of w/c ratios of 0.38 and 0.53 increased by 5% and 25%, respectively. It was reported that the tensile strength decreased by 14% and 6%, respectively, and the modulus of elasticity increased by 3% and 15%, respectively. It was reported that as the coarse aggregate volume increases from 30% to 60% of the total aggregate, the modulus of elasticity increases by 17%.
[19]	lightweight clay aggregate	9.5 12.5 19	river sand	3	0.35 0.40	It was reported that as coarse aggregate dmax increases from 9.5 mm to 19 mm, fracture energy increases by 47.7% and 86.7% for w/c ratios of 0.35 and 0.4, respectively. It was reported that as dmax decreases in mixtures with the same water/cement ratio, fracture toughness also decreases. The opposite trend was observed in the water/cement ratio; As the water/cement ratio increased, the fracture toughness decreased.
[20]	lightweight clay aggregate	10 14 20	Karbala sand	0.1–1	0.33	Increasing aggregate size led to an increase in V hopper flow time and L box height ratio. The best compressive and flexural strengths were observed in mixtures with dmax 10 mm followed by dmax 14 mm. It was reported that water absorbency is generally negatively affected by increasing the aggregate size. It was reported that the drying shrinkage value of mixtures with aggregate dmax 20 mm is lower than other mixtures.
[21]	crushed gravel uncrushed gravel crushed limestone	20	Natural sand	2.41	0.34 0.38	Concrete mixtures made with crushed limestone showed higher strength performance compared to concrete mixtures made with crushed gravel. It was determined that the mechanical performance of SCC mixtures with a maximum gravel grain size of 10 mm is higher than that of mixtures with a maximum gravel grain size of 20 mm.
[11]	Coarse aggregate from Langyang River	-	Fine aggregate from Langyang River	2.82	0.35	It was reported that the fluidity of fresh concrete increases by increasing the fine/coarse aggregate ratio (0.51–0.55). Accordingly, it was emphasized that surface roughness and bonding strength also increased. It was reported that a higher fine/coarse aggregate ratio contributes to more compactness and higher durability of mixtures. However, it was emphasized that mechanical properties depend primarily on the proportion of coarse aggregates. Therefore, despite the increased compactness, it was observed that the increasing fine/coarse aggregate ratio negatively affects the mechanical properties of SCC.
[22]	Dolomite	10 20	natural sand	-	0.32	Three different fine/coarse aggregate ratios were used in the mixtures: 44/56, 47/53, and 50/50. It was reported that the fresh properties of SCC improve as the fine/coarse aggregate ratio decreases. It was determined that the compressive tensile and strength performance is positively affected by the increase in the fine-to-coarse aggregate ratio. The increase in tensile and bending strength was higher than the increase in compressive strength. In terms of the stability and strength of the fresh mix, the study recommends the optimum fine-to-coarse aggregate ratio to be 47% to 53%.

**Table 2:** Chemical composition, physical and mechanical properties of cement and fly ash.

Component	Chemical Content	
	Cement	Fly Ash
SiO_2_	18.86	59.22
Al_2_O_3_	5.71	22.86
Fe_2_O_3_	3.09	6.31
CaO	62.70	3.09
MgO	1.16	1.31
SO_3_	2.39	0.17
Na_2_O + 0.658 K_2_O	0.92	1.4
Cl	0.01	0.001
Specific gravity	3.15	2.31
Loss of ignition	0.52	3.2
Specific surface (cm^2^/g)	3530	4300

**Table 3:** Properties of water-reducing admixture.

Type	Density (g/cm^3^)	pH Value	Chloride Content (%)	Alkaline Ratio, Na_2_O (%)
Polycarboxylate ether based	1.097	3.82	<0.1	<10

**Table 4:** Material rates and quantities used in the production of 1 m³ concrete.

Mix	Cement (kg)	Water/Binder	Fly Ash	Coarse/Fine Aggregate Ratio *	0–5 mm Aggregate (kg)	5–12 mm Aggregate (kg)	Amount of Powder	PCE ** (kg)	Spread (cm)
0.40–15	400	0.40	100	15/85	1432	237	120	2.5	65
0.40–30	400	0.40	100	30/70	1179	473	120	3	67
0.40–45	400	0.40	100	45/55	949	710	120	2.8	66
0.40–60	400	0.40	100	60/40	690	946	120	2.4	67
0.44–15	400	0.44	100	15/85	1383	228	120	3.35	66.5
0.44–30	400	0.44	100	30/70	1139	457	120	3.6	68
0.44–45	400	0.44	100	45/55	916	685	120	3.3	64
0.44–60	400	0.44	100	60/40	666	914	120	3.5	66

* by volume; ** by weight of cement.

**Table 5:** Spreading values, T50, and V-funnel time results of the mixtures.

		Filling Ability	Viscosity/Flowability	
Mixtures	Admixture Dosages * (%)	Spread (cm)	T50 Time (s)	V-Funnel (s)
0.40–15	0.67	66.5	5.86	29.6
0.40–30	0.72	68	3.86	17.67
0.40–45	0.66	64	3.52	16
0.40–60	0.7	65	3.28	16.25
0.44–15	0.5	65	4.8	24
0.44–30	0.6	67	4.3	17.23
0.44–45	0.56	66	3.4	16.44
0.44–60	0.48	67	3.1	19

* by weight of cement.

**Table 6:** Compressive strength, modulus of elasticity, and Poisson’s ratio values.

	28-Day Compressive Strength (MPa)	Modulus of Elasticity (GPa)	Poisson’s Ratio
0.40–15	67.5	30.23	0.129
0.40–30	68.41	34.68	0.116
0.40–45	62.13	33.8	0.103
0.40–60	63.9	30.18	0.1
0.44–15	47.78	20.31	0.058
0.44–30	51.28	22.21	0.139
0.44–45	44.13	24.07	0.117
0.44–60	42.23	25.6	0.063

**Table 7:** Weight losses of the mixture after exposure to temperatures of 300 °C, 600 °C, and 900 °C.

Temperature	105 °C	300 °C		600 °C		900 °C	
Mixtures	Compressive Strength (MPa)	Weight Losses (%)	Relative Strength vs. Compressive Strength at 25 °C (%)	Weight Losses (%)	Relative Strength vs. Compressive Strength at 25 °C (%)	Weight Losses (%)	Relative Strength vs. Compressive Strength at 25 °C (%)
0.40–15	68.95	4.97	93.28	8.63	64.46	17.19	19.15
0.40–30	69.95	5.11	94.56	9.9	61.89	17.31	15.86
0.40–45	63.04	5.48	94.51	8.9	58.47	18.13	14.16
0.40–60	64.99	5.55	96.51	8.41	57.64	16.21	17.84
0.44–15	47.25	5.18	95.75	7.19	69.21	17.17	17.05
0.44–30	51.10	4.99	104.55	8.07	67.52	20.21	15.92
0.44–45	43.24	5.39	98.53	7.06	64.91	18.79	18.92
0.44–60	41.13	6.39	105.15	7.54	61.48	16.56	15.36

## Data Availability

The data used within the scope of the study are given in the text.
